# In Vitro/Ex Vivo Models for the Study of Ischemia Reperfusion Injury during Kidney Perfusion

**DOI:** 10.3390/ijms21218156

**Published:** 2020-10-31

**Authors:** Sebastien Giraud, Raphaël Thuillier, Jérome Cau, Thierry Hauet

**Affiliations:** 1 INSERM U1082 (IRTOMIT), F-86000 Poitiers, France; giraudseb@yahoo.fr (S.G.); raphael.thuillier@univ-poitiers.fr (R.T.); cau.jerome@gmail.com (J.C.); 2 Service de Biochimie, CHU Poitiers, F-86000 Poitiers, France; 3 Faculté de Médecine et Pharmacie, Université de Poitiers, F-86000 Poitiers, France; 4 Laboratoire d’Anatomie, CHU Poitiers, F-86000 Poitiers, France; 5 IBiSA ‘plate-forme MOdélisation Préclinique-Innovations Chirurgicale et Technologique (MOPICT), Domaine Expérimental du Magneraud, F-17700 Surgères, France; 6 FHU SUPORT ‘SUrvival oPtimization in ORgan Transplantation’, F-86000 Poitiers, France

**Keywords:** kidney, in vitro, oxidative stress, perfusion, oxygenation

## Abstract

Oxidative stress is a key element of ischemia–reperfusion injury, occurring during kidney preservation and transplantation. Current options for kidney graft preservation prior to transplantation are static cold storage (CS) and hypothermic machine perfusion (HMP), the latter demonstrating clear improvement of preservation quality, particularly for marginal donors, such as extended criteria donors (ECDs) and donation after circulatory death (DCDs). Nevertheless, complications still exist, fostering the need to improve kidney preservation. This review highlights the most promising avenues of in kidney perfusion improvement on two critical aspects: ex vivo and in vitro evaluation.

## 1. Introduction

Kidney transplantation is the treatment of choice for end-stage kidney failure. However, during this process, kidneys are subjected to ischemia–reperfusion (IR) injury (IRI), involving strong oxidative stress [1], which can be tempered only by improving organ preservation. This is particularly important nowadays, as the donor organ shortage leads the transplant community to accept high-risk organs, like from extended criteria donors (ECDs) and donors after circulatory death (DCDs), which are more sensitive to IRI. Hence, one of the current challenges of the transplant community is to improve preservation quality to limit IRI.

Hypothermic machine perfusion (HMP) has been shown to be the best preservation alternative before kidney transplantation, particularly for fragile organs [2], and especially via maintenance of endothelial function. Our team demonstrated, in a pig kidney model, that the benefits of HMP are mediated by the improvement of cortical microcirculation through the shear stress-induced endothelium release of nitric oxide (NO), due to activated endothelial NO synthase (eNOS) phosphorylation [3]. Indeed, flow cessation triggers endothelial dysfunction during the cold storage of organs. Furthermore, medico-economic evaluation using a Markov model showed that HMP increased life-years and quality-adjusted life-years while reducing costs [4]. Nevertheless, complications still exist, and current HMP technologies provide limited protection. For instance, HMP limits reactive oxygen species (ROS) production in the context of IR [5,6], but does so only partially [7]. As donor demography continues to evolve and further extension of inclusion criteria is expected, there is a clear need for improved preservation. While kidney transplantation appears to lag behind in terms of incorporating new technologies, other organs, such as lungs, liver, and soon the heart, are leading the way. Indeed, for these organs there is no alternative to transplantation. Even though dialysis remains an acceptable substitute, the kidney is the most transplanted organ, and donor demographic changes have been imposing an upgrade in preservation protocols. Better understanding of kidney perfusion mechanisms in the context of IR is needed to identify targets and develop therapies to limit graft injury, especially injury due to oxidative stress. This review highlights the most promising avenues of in kidney perfusion improvement regarding two critical aspects: ex vivo and in vitro evaluation.

## 2. New Challenges in Ex Vivo Perfusion

Currently, the most promising areas in ex vivo perfusion are oxygenation and temperature modulation, as well as the development of protocols combining the two.

### 2.1. Oxygen

During ischemia, oxygen deficiency decreases cellular adenosine triphosphate (ATP) levels, resulting in inhibition of Na^+^/K^+^ pumps and cellular mechanisms and leading to acidosis. Mitochondrial imbalance and ROS production then pave the way for severe lesions during reperfusion, with long-term effects in terms of graft survival [8].

In our hands, using a DCD model of porcine kidney HMP, we observed a rapid decline in perfusion solution oxygen pressure 10 min after the start of perfusion, from 150 mmHg to 6.8 ± 1.4 mmHg in 200 min. Similarly, renal cortical oxygen pressure descended from 10.2 ± 8.7 mmHg to zero within 60 min, implying an initial oxygen consumption (QO_2_) rate at 3.4 ± 1.2 mmol/min per 100 g, which then decreased to 0.0–0.2 at 120 min [9]. These findings place oxygen supply at the top of the list for HMP improvement.

Several solutions exist, as discussed below, and it should be noted that oxygen is a double-edged sword, insofar as over-oxygenation can promote ROS production [10]. It is consequently of paramount importance to properly study key parameters of concentration, method, and timing of delivery.

#### 2.1.1. Gaseous Oxygen Supply

Several studies have evaluated the potential benefits of active oxygenation. Thuillier et al., showed that porcine kidneys preserved in HMP with 100% O_2_ had better kidney graft function recovery, and reduced levels of interstitial fibrosis compared with kidneys preserved in HMP without O_2_ [11]. Darius et al., demonstrated that preservation of porcine kidney in HMP with 95% O_2_ permitted a faster increase in renal flow during preservation and a faster kidney recovery at reperfusion [12]. Hoyer et al., confirmed that porcine kidneys preserved in HMP with 100% O_2_ had better creatinine clearance [13]. Recently, Patel et al., have shown that HMP of porcine kidneys with 95% O_2_ led to significantly improved metabolic profiles (lower lactate, higher glutamate, higher ATP) compared with HMP with 21% O_2_ [7]. Kasil et al., demonstrated that preservation by HMP with 100% O_2_ tended to improve kidney perfusion, and thereby decrease renal resistance during HMP [14]. As these preclinical results were promising, clinical studies have been initiated, such as the ongoing clinical trial evaluating oxygenated HMP by the Consortium for Organ Preservation in Europe (COPE; COMPARE Trial, ISRCTN32967929) (Table 1).

Intervention time for oxygenation during kidney preservation is a critical step. Several studies showed that short-term hypothermic oxygenation after the preservation period (HOPE) is beneficial, with reduction in the incidence of tubular damage and better kidney function [15]. Currently, clinical studies are designed to (i) explore the effects of HOPE on ECD kidney allografts donated after brain death (NCT03378817), or (ii) to compare end-ischemic HMP with or without O_2_ = in ECD kidneys (POMP Trial, ISRCTN63852508) (cf. Table 1).

#### 2.1.2. Oxygen Carriers

As gaseous perfusion imposes technological imperatives, and may not be compatible with current legislation on oxygen transport, the need for oxygen carriers has become evident. As red blood cells (RBCs) are not compatible with hypothermia and the mechanical device itself, it appears that the ideal oxygen carrier should be cell-free. Several carriers have been developed.

Artificial oxygen carriers (AOCs) have been developed to compensate for a shortage of blood products. However, most research on AOCs is in the field of blood substitutes, and is not being pursued for the purposes of organ preservation.

Hemoglobin-based oxygen carrier (HBOC)-201 is a bovine-derived free hemoglobin (Hb) (Hemopure; HBOC-201) obtained by glutaraldehyde polymerization. HBOC-201 has been evaluated in ex vivo perfused porcine kidney, and demonstrated similar renal function and equivalent tissue oxygen saturation compared to a blood-treated kidney [16]. This molecule has also been evaluated in gradual rewarming protocols (see below), where it demonstrated the ability to withstand a wide range of temperatures [17].

HbV is a human-derived hemoglobin encapsulated in phospholipid vesicles enhanced by polyethylene glycol (PEG) conjugation, which is characterized by the absence of antigens, and a smaller size than red blood cells enabling penetration through constricted vessels and a longer shelf life [18].

Rabbit model studies have demonstrated that HMP supplemented with oxygen-carrying hemoglobin or polyethylene-glycolated bovine hemoglobin (bHb-PEG) could extend preservation times and decrease ischemic injury [19]. Furthermore, conjugation with PEG chains effectively increases the circulation time of Hb and avoids its nephrotoxicity. It can also significantly increase colloidal osmotic pressure and viscosity [20].

PEGylated Hb products (using human adult hemoglobin (HbA) as the original substrate) have been developed as HBOCs for clinical trials. However, as the quantity of HbA available from donations may be insufficient for mass production of HBOCs, use of an alternative Hb, such as bovine hemoglobin (bHb), is necessary. Wang et al., showed that bHb-PEG is expected to function as a potent HBOC due to its high oxygen delivery and strong plasma-expanding ability [20].

The medical device HEMO_2_ life contains a natural extracellular hemoglobin (M101) isolated from marine lugworm (*Arenicola marina*), with a high affinity to oxygen, which is able to specifically deliver oxygen to hypoxic tissues. Thuillier et al. showed that the addition of M101 in preservation solution during CS improves graft renal function [21]. Moreover, Kaminski et al., demonstrated that M101 was compatible with HMP and preserved tissue ATP contents, improving short- and long-term functional outcomes, as well as tissue integrity [22].

In a recent multicentric human clinical trials using CS and HMP in kidney transplantation from deceased donors, M101 has proved its safety and showed promising results in improving kidney transplantation outcomes (OxyOp; NCT02652520) [23]. It represents an important advance as an oxygen carrier in kidney transplantation.

Novel approaches are also in development: ErythroMer, which offers pH-dependent oxygen uptake/release control using a 2,3-diphosphoglycerate transporter [24]; and hemerythrin, another transporter issued from marine invertebrates that is modifiable with polyethylene glycol and glutaraldehyde to improve its performance [25].

While each approach appears to have specific advantages—e.g., HBOC has a lengthy history, PEGylated Hb offers the benefits of PEGs, and M101 presents naturally extracellular properties and a wide range of functional temperatures—the lack of common test models and protocols renders an accurate comparison difficult. A comparative study would be the best way to properly benchmark these molecules, preferably in models allowing easy transfer of the results to the clinic.

These results show that oxygenation during preservation is beneficial, through either of the routes highlighted above. Hence, the risk of hyperoxia and subsequent ROS generation appears minimal. The possible additivity of combining both approaches was tested in a recent study of kidney transplantation by Kasil et al., using gaseous oxygenation with an oxygen carrier (M101 in this case) during HMP [14]. Following transplanted animals for 3 months, the authors highlighted the benefits of oxygenation, but without demonstrating additivity between the two approaches.

Such an approach could be combined with antioxidant therapy, which can protect against oxidative damage induced by IR in the context of kidney transplantation.

### 2.2. Alternative Preservation Temperatures

Hypothermia, currently used for preservation, is being questioned by the scientific community. Numerous articles highlight its worsening of ischemic injuries through mechanisms like the reduction of ATP metabolism activity and mitochondrial activity in general, as well as the reduction of membrane ion transport, inducing osmotic perturbation, increased endothelial activation, and the solidification of lipid membranes [26]. Indeed, dramatically lowered temperature decreases mitochondrial oxygen consumption, and also increases kidney and in vitro cell lipid peroxidation, due to a loss of ROS scavenging in the cold [27]. As hypothermia associated with oxygen deprivation induces oxidative stress and the loss of ROS scavenging [27], increased preservation temperature could help to reduce oxidative stress and overall kidney injury.

Recent studies have advocated the use of room-temperature or more physiological temperature, which might potentially diminish injuries related to hypothermia [28]. Indeed, controlled oxygenated rewarming (COR), sub-normothermic machine perfusion (SMP) and ex vivo normothermic machine perfusion (NMP) are ex vivo alternatives that might potentially diminish injury-related hypothermia.

Controlled oxygenated rewarming (COR) represents a brief and controlled return to 20 °C after standard CS. It provides a gradual adaptation of energy metabolism, counteracting rewarming injury [29], and is more protective than oxygenated HMP post-CS or continuous oxygenated HMP [30]. COR reduces ROS production, improves mitochondrial and aerobic efficiency, enhances kidney function compared to NMP, and improves post-transplant graft outcome compared to CS [31]. As clinical feasibility is demonstrated [32], COR appears to be an alternative post CS compared to NMP [29,33].

Sub-normothermic machine perfusion (SMP) is aimed at protecting the organ from cold ischemia injury without increasing the metabolism to a level where oxygen supply is needed. Tissier et al. showed that, in rabbit hearts, as in isolated rat cardiomyocytes, per-ischemic ROS generation dramatically decreased at 32 versus 38 °C [34]. Hoyer et al. showed that SMP led to significantly higher blood flow and urine output versus oxygenated HMP [35]. Brasile et al. demonstrated the feasibility of maintaining human kidneys with exsanguinous metabolic support (EMS) at 32 °C for up to 48 h [36]. However, these SMP technologies have not yet reached the clinic.

NMP (also called ex vivo normothermic machine perfusion (EVNP)) is aimed at restoring physiological cellular metabolism/activity [37]. It maintains cellular processes at physiological temperature prior to transplantation by circulating a warm, oxygenated red cell-based solution into the kidney. The red cell-based perfusate was designed to reduce the likelihood of inflammation and oxidative injury, as well as to improve oxygen distribution [38]. While technically more complex, its advantages include the reduction of cold ischemia, as well as increased metabolic activity, while providing options to assess renal function and modify/repair grafts prior to transplantation. In the second millennium, this technology was widely tested in Europe, Canada, and United States [39], demonstrating better function and regeneration compared to CS [40] or HMP [41]. Hamar et al. demonstrated that a porcine kidney exposed to 30 min of warm ischemia (WI) + 8 h EVNP had lower daily serum creatinine levels and better regeneration markers compared to grafts exposed to WI + 8 h CS [40]. Metcalfe et al. observed superior graft function with porcine kidneys at 38 °C during 16 h of autologous blood perfusion versus HMP [41]. Moreover, prolonged EVNP duration (16 h versus 8 h) is beneficial for graft function recovery [42]. Indeed, Fabry et al. demonstrated that cold pre-flush prior to EVNP aggravates ischemia reperfusion injury compared to direct EVNP [33], suggesting that it should be performed throughout the preservation period. Such experimental animal data encouraged translation of EVNP to the clinic [43]. EVNP has recently been introduced into clinical practice, and early experience of renal transplantation after EVNP shows that the technique is feasible, safe, improves early graft function, and is superior to CS [38,44,45,46,47,48,49]. Weissenbacher et al. showed that EVNP of human kidneys for 24 h did not affect tissue integrity and permitted safe urine production [44]. Hosgood et al. demonstrated that brief (35 min to 63 min) EVNP following CS improved renal human kidney function after allo-transplantation, with a reduction of DGF versus CS and no difference in graft or patient survival at 12 months. They suggested an interesting resuscitation role of EVNP for kidneys previously deemed unsuitable for transplantation [49]. Hameed et al. demonstrated that EVNP of discarded marginal kidneys induced significant mechanistic benefits compared to CS alone [50]. EVNP improved kidney function, renal blood flow, and resistance over the course of perfusion. It also increased urine output and oxygen consumption, restored depleted ATP levels, resolved nonperfused kidney regions, and upregulated pathways promoting cell survival and proliferation. Finally, it reduced oxidative stress and apoptosis markers, and globally reduced IRI.

Increased metabolic activity implies increased oxygen requirements and oxygen consumption, and the EVNP process consequently requires an adequate continuous oxygen supply [51], typically delivered by packed RBCs. However, this has several potential disadvantages, including use of a scarce precious resource, infectious transmission, RBC hemolysis, and logistical difficulties associated with cross-matched blood. In this light, use of an alternative oxygen carrier could be judicious. This approach is feasible, as shown in the liver, where perfusing with HBOC-201 is similar to perfusion with RBC and FFP [52]. Laing et al. demonstrated that Hemopure (an HBOC) can be used as an alternative oxygen carrier in NMP, and replace RBCs. Hemopure may be logistically, rheologically, and immunologically superior to packed RBCs when used in NMP [53]. Aburawi et al. demonstrated that use of a synthetic HBOC solution can offer a logistically more convenient off-the-shelf alternative to packed RBCs in NMP [54]. This approach is being tested in a clinical trial on marginal livers using sequential hypo- and normothermic reperfusion, in which oxygen delivery is facilitated by using an HBOC (DHOPE-COR-NMP trial, NTR5972) (Table 1). Future results could be transposable to kidney NMP. Furthermore, at the American Transplant Congress 2019, Detelich et al. presented another alternative, with an oxygenated, cell-free perfusate (Williams E-based perfusate oxygenated with a gas mixture of 95% O_2_/5% CO_2_) for up to 6 h in human kidney EVNP. This resulted in stable hemodynamics, a sufficient supply of O2 for aerobic metabolism, and similar ATP level restoration compared to RBC-based perfusate.

As cold ischemia time remains one of the major risk factors for kidney dysfunction, NMP seems to be a major alternative [55], which it already represents for liver [56]. Moreover, an NMP circuit provides the necessary conduit for cell therapy application or pharmacological treatment prior to organ transplantation. This NMP technique is currently being evaluated in a United Kingdom-based, phase II, multicenter, randomised controlled trial to assess the efficacy of EVNP in DCD kidney transplantation (ISRCTN15821205) (Table 1).

However, the study of oxygenation, antioxidant therapy, and temperature modulation should ideally be carried out with in vitro cellular models, in order to best define the cell-specific dose/timing protocols, which will then be evaluated in ex vivo and in vivo models.

## 3. In Vitro Perfusion Models

Blood flow-induced shear stress is one of the main regulators of endothelial cell phenotype, function, and metabolism [57,58]. It is regulated by the microenvironment and rheological behavior of the perfusion medium [59]. As a result, physiologically relevant in vitro studies of vascular cells require realistic environments. The objective would be to have an in vitro perfusion system to mimic graft perfusion at the cellular scale on a population, such as endothelial cells—primary targets of IRI. Indeed, development of an in vitro system with control over shear stress, O_2_ levels, and perfusate composition would advance the understanding and predictability of human endothelial functionality in healthy and ischemia–reperfusion conditions. The setting up and control of an in vitro perfusion model allows study of the mechanistic impact of flow modulation on endothelial cells, mimicking the “Flow” or “no flow” periods undergone by the graft between the collection and transplantation. In vitro cell culture under flow is more physiological for standard in vitro static culture for endothelial cells, lymphatic endothelial cells, and nephron-epithelial cells, which are naturally under flow in physiological situations. As a result, to mimic ischemic extracorporeal perfusion preservation or reperfusion, in vitro models have been developed. These take into account physiological shear stress values (in dyn/cm^2^), which depend on pressure, vessel size, vessel type (artery, vein, and capillary), tissue, and size of the animal [60,61]. These models sometimes include flow type control, e.g., laminar flow (in many vessels), pulsatile laminar flow (caused by heartbeats in large vessels), or perturbated flow (in the case of pathology).

**Figure 1:**
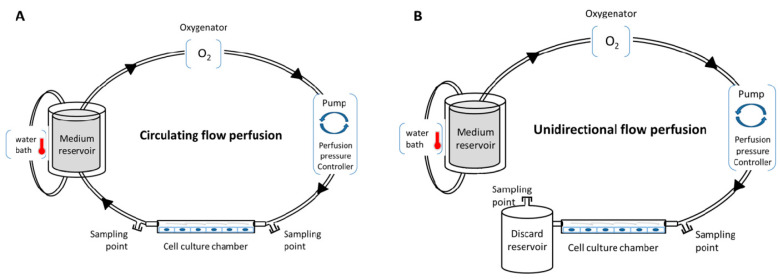
Conventional in vitro perfusion models. (**A**) Circulating flow perfusion design with closed circulation; (**B**) unidirectional flow perfusion design without recirculation.

Various in vitro perfusion systems exist, such as an IBIDI pump system, Cytodyne, FlexCell, or Stovall Flow Cell. These systems allow users to regulate fluid shear stress to cells in culture with laminar, pulsatile, or oscillating flow. These systems use a computer-controlled peristaltic pump to regulate in real time the flow frequency/pressure, and to induce various ranges of shear stress, with either circulating flow (closed circulation of the medium) or unidirectional flow (Figure 1). In addition, they are compatible with oxygenator and heating systems for temperature modulations, and propose several channels with different geometries and volumes to induce a specific shear stress range. This provides a robust basis for repeatability experiments with total control of all perfusion parameters, enabling the exploration of cell (or co-cultured cell) physiology, cell function [57,61,62], secretome, and the metabolic impact of the parameters (temperature, oxygen, medium, etc). Several applications can be performed with these systems: (i) investigating the influence of shear stress on cell physiology and morphology with various experimental assays, such as proliferation/death test, immunofluorescence, flow cytometry, metabolic, proteomic and transcriptomic, on the cultured cells; (ii) investigating functional tests, such as leukocyte rolling and adhesion assays, or cell migration or transmigration assays; (iii) investigating the perfused medium with biochemical tests, enzyme-linked immunosorbent assay (ELISA), and metabolomic assay; and (iv) impedance measurements under flow stimulation (cells could be cultivated in channels with electrode arrays) [63]. Compatibility with high-throughput biotechnologies allows better understanding of the cellular and molecular networks that occur in cells under perfusion.

Such in vitro perfusion systems could permit in-depth evaluation of cell physiology when subjected to organ donor/recipient pathologies (such as hypertension and diabetes) and to ischemia-reperfusion injury (with variation of flow, oxygen level, temperature, and extracellular medium composition).

Such a system has been shown to be a more physiological microfluidic integrative model for endothelium evaluation, compared to conventional culture condition [61], reducing pro-apoptotic and angiogenic gene expression, upregulating Kruppel-like factor 2 (KLF2) and eNOS gene expression, limiting proliferation, and improving formation of continuous barriers, compared to static culture condition [57].

These systems could elucidate the mechanisms controlling vessel plasticity [64] and endothelial phenotype and permeability [65], which are particularly impacted during IRI. For instance, a study of an in vitro hypertension model showed the mechanisms through which a highly cyclic stretch induced abnormal endothelial cell proliferation [66]. Another study explored the context of diabetes on perivascular cell recruitment [64]. Moreover, these perfusion systems offer the possibility of co-cultured cells. It is of utmost interest to study leukocyte rolling and adhesion on a monolayer of endothelial cells, which have a major role in the IRI. Indeed, IR induces leukocyte infiltration in the interstitial compartment, where the activated leukocytes release toxic ROS. It is known that shear stress is an important regulator of leukocyte adhesion dynamics and inflammatory response. In this case, using a parallel plate-flow chamber (PPLC), and especially a co-cultured Transwell flow chamber, functioned better than most experimental approaches for leukocyte adhesion and diapedesis studies [67]. It has been demonstrated that oxygen-regulated intercellular adhesion molecule 1 (ICAM-1) and vascular cell adhesion protein 1 (VCAM-1) expression and neutrophil adhesion on in vitro endothelial cells cultured under perfusion. In addition, oxygen regulated hydrogen peroxide release from neutrophils [68]. In addition, these systems render it possible to evaluate cell-secreted markers, such as alarmins, cytokines, chemokines, growth factors, hormones, and ROS, which play a pivotal signaling role during IRI.

It is well-established that hypoxic conditions (O_2_ < 5%) alter endothelial phenotypes and permeability [65]. Interestingly, the impact of oxygen tension has also been studied on such models [69], showing the combined influence of flow and oxygen on endothelial cell phenotype, apoptosis, proliferation, adhesion, and gene expression [57,68]. The underlying mechanisms involve key regulators of cell signaling, particularly O_2_ and the eNOS/NO pathway [70] involving KLF2 [71]: in vitro laminar flow on endothelial cells induced KLF2 upregulation and its downstream effectors (eNOS and thrombomodulin), promoting NO production. KLF2 acts selectively as an important mechano-activated transcription factor in multiple endothelial functions, like the regulation of endothelial transcriptional programs controlling vascular tone, blood vessel development, thrombosis/hemostasis, and inflammation [58]. Shear stress was demonstrated to be one of the protective mechanisms of perfusion, through a mechanism which was thought to involve KLF2 and eNOS/NO expression in large animal models (porcine kidney) [72], small animal models (rabbit kidney) [73], and in vitro models of endothelial cells [74]. These results are in association with decreased KLF2 and eNOS expression, both in vitro and in vivo, due to flow cessation in the ischemia model [57,75].

However, a large animal study showed that cortical KLF2 and eNOS levels were not affected by HMP [3]. Indeed, HMP increased eNOS activation (phosphorylation), and thus NO production through AMP-activated protein kinase (AMPK), concomitant with an increase of cortical microcirculation after reperfusion in vivo [3], highlighting the importance of thorough studies, including diverse and adapted model types [76].

In vitro models have also demonstrated the involvement of shear stress and oxygenation in antioxidant response, through the nuclear factor (erythroid-derived 2)-like 2/heme oxygenase 1/nicotinamide adenine dinucleotide phosphate dehydrogenase (NRF2/HO-1/NAD(P)H dehydrogenase) pathway [77], with HO-1 regulating the directional migration of endothelial cells [78] and shear stress regulating HO-1 through statin concentration reduction [74]. Hence, KLF2 and NRF2 are central to laminar shear stress-mediated regulation of the antioxidant response [79], with NRF2 playing an important role in the angiogenic capacity of endothelial cells, particularly under oxidative stress [80]. Finally, antioxidants can be combined with shear stress to upregulate KLF2, eNOS, and angiopoietin 2 (ANG2) gene expression [57].

Such in vitro perfused models could explore the benefits of antioxidant drugs and oxygen carriers, such as HEMOXCell (a marine oxygen carrier having properties of high oxygen sensitivity, to be used as an oxygen additive during cell culture), which increased the growth rate of cells [81]; HBOC, which in dynamic cell culture impacts the endothelial phenotype, as well as the eNOS/NO and HO-1 pathways, and reduces oxidative stress levels [82]. Also, it seems to be essential to include shear stress on in vitro assays to improve our understanding of the effects of antioxidant drugs and oxygen carriers on endothelial cells.

It appears that the use of oxygen must take into account the need for antioxidant strategies, as oxygenation is a double-edged sword: on the one hand, it provides the essential gas for tetra-reduction, leading to ATP production in the mitochondria, while on the other hand it provides the same molecule that can easily be mono-reduced to produce ROS. Hence, compensating for the eventual increase in ROS production by careful use of antioxidants, preferably targeting major ROS production sites like the mitochondria, appears to be a wise complement to oxygenation.

**Figure 2:**
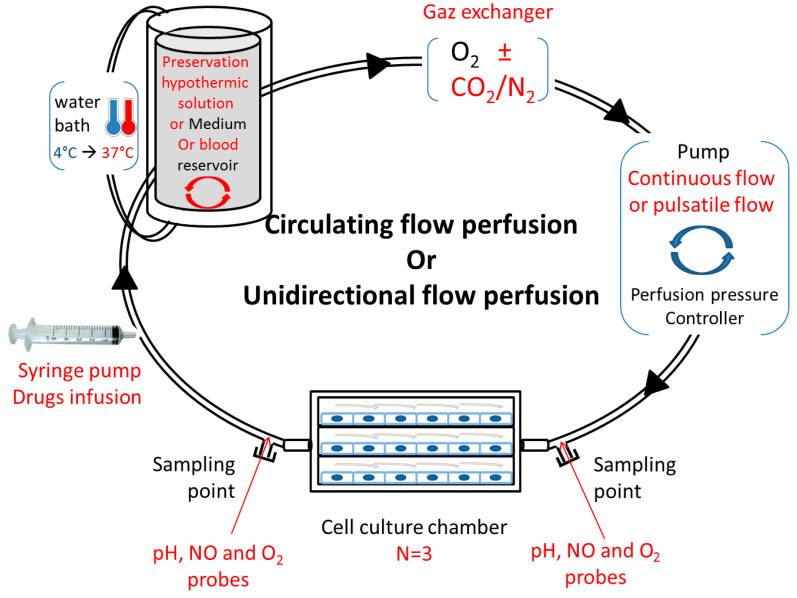
Proposition for an adapted in vitro, physiological ischemia–reperfusion model. Circulating or unidirectional flow perfusion design with adapted materials (red texts) for in vitro ischemia–reperfusion study.

Studying vascular disease, such as ischemia–reperfusion, in conventional static cell cultures does not effectively mimic the complex vascular microenvironment, and may not accurately predict in vivo vascular injury responses. These in vitro perfusion systems offer opportunities to study and predict the cell metabolism and phenotype in healthy, pathological, and drug-treated environments. It is interesting to study the impact of oxygenation and temperatures on cell quality and metabolism, and it is also necessary to include approaches closer to the physiological conditions of the whole tissue or organ. It is likewise important to design an in vitro perfusion model in adequation with the new research fields in kidney preservation. Several implementations could be considered: a syringe pump for cell or drug infusion, a water bath from 4 °C to 37 °C, a reservoir with a “mixer” for various perfusate compositions (preservation solution or blood), a gas exchanger compatible with several compositions, a pump coupled to a controller to generate a continuous or pulsatile flow, or sampling points on both sides of the multi-channel flow chamber (Figure 2). The whole mechanism is ideally coupled to a computational tool.

However, these in vitro perfusion systems should be integrated in a multiple model investigation, as some results do not fully correlate with more complex models [76,83]. Such an analytic pipeline could be further improved with the use of organoids, which are mini-organs derived from induced pluripotent stem cells (iPSCs) [84]. Their size permits their use in in vitro, three-dimensional (3D), perfusable chip systems, providing a versatile platform for regenerative and personalized medicine, understanding of disease models, and drug evaluation [85,86]. Indeed, 3D cell cultures (for a single-cell population, co-culture, or organoides) are in vitro models, with more realistic representations of a biological tissue, representing a significant improvement compared to traditional monolayers.

Another interest is the compatibility of these in vitro perfusion systems with a computational tool to model cell–cell, cell–surface, and cell–medium interactions in an in silico mathematical model. This in silico study by computational tool is compatible with single-cell population cultures, co-culture model [87], or 3D microenvironment [88]. Indeed, to comprehend and predict such complex cell culture phenomena, and in designing of optimised cell culture conditions, mathematical modelling and numerical simulations are effective strategies [89]. Developing a comprehensive and predictive in silico model is a major interest, and should contribute to the field of integrated in silico/in vitro analysis of biological systems. The predictions made by these in silico simulations will be useful for optimising in vitro evaluations and limiting in vivo studies (in compliance with the 3Rs rule (Replacement, Reduction and Refinement)).

## 4. Conclusions

Nowadays, organ preservation is entering in the era of dynamic intervention. The quality of marginal organ preservation needs to be improved, as HMP alone is not sufficient to decrease IRI and complications. Superior outcomes can be achieved through better management of oxygenation and temperature, and several options exist to prepare graft more physiologically, with adequate technology and adapted in vitro and ex vivo methods. Interestingly, there are ways to adapt such improvements to existing perfusion infrastructures, which could speed up the translation of observed improvements to the clinic. However, more logistically challenging options present undeniable advantages, with an opportunity for functional monitoring, therapeutic intervention, and repair. Hence, future perfusion protocols should be tailored for the specific needs of grafts of inferior quality, which can be achieved via the development of cellular and organ perfusion labs, in order to centralise the evaluation and reconditioning of marginal graft prior to transplantation. In this setting as well, priority organs are leading the way, but the decreased quality of donated organs encourages such organisation for the kidney as well, perhaps auguring the creation of multi-organ evaluation platforms.

## Figures and Tables

**Table 1:** Ongoing clinical trials with non-published results.

Number	Acronym	Title	Conditions	Study	Sponsors and Collaborators	Phase
ISRCTN32967929	COPE-COMPARE	Cold oxygenated machine preservation of aged renal donation after cardiovascular death transplants	Injury, occupational diseases, poisoning	A multicentre, double-blind, randomised, parallel-group, paired trial to compare the effect of hypothermic machine perfusion preservation with and without the addition of oxygen in transplantation of Maastricht category III kidneys donated after circulatory death from donors aged 50 years or older	Abdominal Transplant Surgery, University Hospitals Leuven, Belgium; University of Oxford, United Kingdom	N/A
NCT03378817	N/A	Hypothermic oxygenated machine perfusion of extended criteria kidney allografts from brain death donors	Reperfusion injury, hypothermic oxygenated machine perfusion	The present trial is an investigator-initiated pilot study on the effects of hypothermic oxygenated perfusion (HOPE) on expanded criteria donor (ECD) allografts in donation after brain death (DBD) kidney transplantation. Fifteen kidney allografts will be submitted to 2 h of HOPE before implantation, and are going to be compared to a case-matched group transplanted after conventional cold storage (CCS).	University Hospital, Aachen, Germany	N/A
ISRCTN63852508	COPE-POMP	COPE-POMP: “in house”, pre-implantation, oxygenated, hypothermic machine perfusion reconditioning after cold storage versus cold storage alone in expanded criteria donor (ECD) kidneys from brain-dead donors	Surgery Machine perfusion preservation techniques for ECD kidneys	A prospective, randomised, parallel group, single-blinded, controlled, multi-center, non-paired superiority trial to compare the effect of short-term “in-house” oxygenated, hypothermic machine perfusion preservation after static cold storage to static cold storage alone in the transplantation of expanded criteria donor (ECD) kidneys donated after brain death	Department of General Visceral and Transplant Surgery, University Hospital Essen, Germany; University of Oxford, United Kingdom	Phase II
ISRCTN15821205	N/A	Improving function of transplanted kidneys	Urological and genital diseases	This is a United Kingdom-based, phase II, multicentre, randomised controlled trial of ex vivo normothermic perfusion versus static cold storage in a donation after circulatory death renal transplantation	Department of Surgery, University of Cambridge; Addenbrooke’s Hospital, Cambridge, United Kingdom	Phase II
NTR5972 (Trial NL5817)	DHOPE-COR-NMP	A single center clinical trial to assess viability of high-risk donor livers using hypothermic and normothermic machine perfusion with rewarming phase prior to transplantation	Normothermic machine preservation, normothermic machine perfusion, ex situ viability testing, liver transplantation	Each high-risk donor liver accepted for this study will undergo machine perfusion. First, dual hypothermic oxygenated machine perfusion (DHOPE) will take place, before gradually rewarming the donor liver to a normothermic temperature. Secondly, after rewarming, NMP will be performed to allow graft assessment.	University Medical Center Groningen; Department of Surgery Groningen, the Netherlands	N/A

## Abbreviations

AMPK	AMP-activated protein kinase
ANG2	Angiopoietin-2
AOCs	Artificial oxygen carriers
ATP	Adenosine triphosphate
bHb	Bovine hemoglobin
COPE	Consortium for Organ Preservation in Europe
COR	Controlled oxygenated rewarming
CS	Cold storage
DCD	Donation after circulatory death
ECD	Extended criteria donor
ELISA	Enzyme-linked immunosorbent assay
EMS	Exsanguinous metabolic support
eNOS	Endothelial NO synthase
EVNP	Ex vivo normothermic perfusion
FFP	Fresh frozen plasma
Hb	Hemoglobin
HbA	Adult hemoglobin
HBOC	Hemoglobin-based oxygen carrier
HMP	Hypothermic machine perfusion
HO-1	Heme oxygenase-1
HOPE	Hypothermic oxygenation after the preservation period
ICAM-1	Intercellular adhesion molecule 1
IR	Ischemia–reperfusion
IRI	Ischemia–reperfusion injury
KLF2	Kruppel-like factor-2
NAD(P)H	Nicotinamide adenine dinucleotide phosphate
NMP	Normothermic machine perfusion
NO	Nitric oxide
NRF2	Nuclear factor (erythroid-derived 2)-like 2
PEG	Polyethylene glycol
PPLC	Parallel plate-flow chamber
PSCs	Pluripotent stem cells
QO_2_	Oxygen consumption
RBC	Red blood cell
ROS	Reactive oxygen species
SMP	Sub-normothermic machine perfusion
VCAM-1	Vascular cell adhesion protein 1
